# Human Immunodeficiency Virus Envelope Protein Gp120 Induces Proliferation but Not Apoptosis in Osteoblasts at Physiologic Concentrations

**DOI:** 10.1371/journal.pone.0024876

**Published:** 2011-09-12

**Authors:** Nathan W. Cummins, Anna Klicpera, Amy M. Sainski, Gary D. Bren, Sundeep Khosla, Jennifer J. Westendorf, Andrew D. Badley

**Affiliations:** 1 Division of Infectious Disease, Mayo Clinic, Rochester, Minnesota, United States of America; 2 Program in Translational Immunovirology and Biodefense, Mayo Clinic, Rochester, Minnesota, United States of America; 3 Department of Molecular Pharmacology and Experimental Therapeutics, Mayo Clinic, Rochester, Minnesota, United States of America; 4 Division of Endocrinology, Diabetes, Metabolism and Nutrition, Mayo Clinic, Rochester, Minnesota, United States of America; 5 Department of Orthopedic Surgery, Mayo Clinic, Rochester, Minnesota, United States of America; University of Cape Town, South Africa

## Abstract

Patients with HIV infection have decreased numbers of osteoblasts, decreased bone mineral density and increased risk of fracture compared to uninfected patients; however, the molecular mechanisms behind these associations remain unclear. We questioned whether Gp120, a component of the envelope protein of HIV capable of inducing apoptosis in many cell types, is able to induce cell death in bone-forming osteoblasts. We show that treatment of immortalized osteoblast-like cells and primary human osteoblasts with exogenous Gp120 *in vitro* at physiologic concentrations does not result in apoptosis. Instead, in the osteoblast-like U2OS cell line, cells expressing CXCR4, a receptor for Gp120, had increased proliferation when treated with Gp120 compared to control (P<0.05), which was inhibited by pretreatment with a CXCR4 inhibitor and a G-protein inhibitor. This suggests that Gp120 is not an inducer of apoptosis in human osteoblasts and likely does not directly contribute to osteoporosis in infected patients by this mechanism.

## Introduction

Prior to the advent of highly active antiretroviral therapy (HAART), HIV infection was considered to be ultimately fatal. However, administration of HAART, particularly in resource-rich settings, has dramatically changed the clinical course of the disease, with patients newly infected with HIV now expected to have a near normal life expectancy. With this increased life expectancy, though, has come the recognition of many long term consequences of chronic HIV infection, including insulin resistance and the metabolic syndrome, accelerated cardiovascular disease, and increased risk of malignancy, that are typically associated with aging [1]. HIV-infected patients also have lower bone mineral density, and higher prevalences of both osteopenia and osteoporosis, compared to age and sex matched HIV-uninfected controls [2]–[4]. This translates into an increased risk of fracture and decreased rate of fracture healing [5], [6]. The cause of the increased bone disease seen in HIV infection, however, remains unclear.

Osteopenia and osteoporosis generally result from an uncoupling of the homeostatic balance between bone resorbing osteoclasts and bone forming osteoblasts [7]. Several mechanisms have been proposed to account for the increased bone disease seen in HIV infected patients. Traditional risk factors for osteoporosis, including low body mass index, inactivity, and tobacco and alcohol use, are over-represented in the HIV-infected population. Other contributing factors seen in chronically HIV-infected patients include Vitamin D deficiency, hypogonadism, and hyperparathyroidism secondary to chronic renal insufficiency [3], [4]. Antiretroviral therapy itself, and particular medications such as tenofovir and protease inhibitors, may contribute to bone disease, either indirectly through renal phosphorous wasting and hyperparathyroidism in the case of tenofovir, or directly through stimulation of osteoclastogenesis by protease inhibitors [8]–[10]. The chronic immune activation and elevated levels of pro-inflammatory cytokines seen in HIV infection, particular tumor necrosis factor α (TNFα), interleukin 6 (IL-6) and RANKL, may stimulate osteoclastogenesis and osteoclast activity [11], [12]. However, there have been few studies investigating the direct effect of HIV-infection on bone forming osteoblasts. Histomorphometric examination of bone biopsy samples from HIV-infected patients reveal decreased number and activity of osteoblasts compared to uninfected controls [13]. However, human osteoblasts are unable to be infected with the HIV virus, likely due to low level expression of the cell surface receptor CD4 required for cell attachment and entry [14]–[15].

Several investigators have questioned whether HIV-associated proteins have direct effects on osteoblast number or function. Gp120 is the extracellular component (in conjuction with the transmembrane Gp41) of the Gp160 HIV envelope protein, and is expressed on the surface of infective HIV virions and HIV-infected cells. Gp120 on virions binds to the CD4 cell surface receptor, and when engaged with one of two cellular co-receptors (CCR5 or CXCR4), leads to membrane fusion of the virus and host cell and consequent cell entry. Later in the life cycle of the virus, Gp120 is expressed on the cellular membrane and is incorporated into the membrane of the budding virus. Gp120 can be detected in the serum and extracellular tissue matrices of HIV-infected patients, even in the presence of suppressive antiretroviral therapy [16]. Therefore, Gp120 would make a logical candidate for a protein-induced pathologic effect on osteoblasts in HIV infection. Exogenous treatment of osteoblasts with the HIV envelope glycoprotein Gp120 or p55-gag protein decreases osteoblast function, as measured by calcium deposition and alkaline phosphatase expression, through a decrease in RUNX2 and an increase in PPARγ expression [17]. Furthermore, Gp120 induces apoptosis in a number of cell types, including CD4 T lymphocytes, neurons, hepatocytes, and cardiomyocytes [18]. Gibellini et al demonstrated that inactivated HIV virus and recombinant Gp120 were able to induce apoptosis in primary human osteoblasts and an immortalized osteoblast-like cell line (HOBIT) *in vitro* through a mechanism that is partially inhibited by blocking antibodies to TNFα [19].

In this investigation, we sought to confirm a cytotoxic effect of Gp120 on osteoblasts *in vitro*, and to determine which cellular receptor(s) (CD4, CCR5 or CXCR4) and signaling mechanism(s) are necessary for Gp120-induced apoptosis of osteoblasts. This information could have direct implications on developing therapies to prevent or reverse the accelerated bone disease seen in HIV-infected patients. On the contrary, our results suggest that Gp120 is not directly cytotoxic to human osteoblast-like cells, and may actually induce proliferation in a small proportion of osteoblast-like osteosarcoma cells through engagement with CXCR4.

## Methods

### Cell Lines and Culture

Jurkat T cells (American Type Culture Collection, Manassas, VA) were maintained in RPMI medium (Mediatech, Inc, Manassas, VA) supplemented with 10% heat-inactivated fetal calf serum (FCS) and 2 mM L-glutamine, and passed twice weekly. Human osteoblast-like cell lines, including fetal osteoblasts (FOB) [20], human osteoblast-like SV-40T antigen immortalized cell line (HOBIT) [21], and the U2OS osteosarcoma cell line [22], were maintained in DMEM/F12 medium (Gibco, Carlsbad, CA) supplemented with 10% heat-inactivated fetal calf serum and L-glutamine. Primary human osteoblast cultures from two HIV-uninfected donors (HOB-1 and HOB-2) were obtained from Promocell (Heidelberg, Germany) and maintained in the Promocell Osteoblast culture medium. Osteoblast cultures were passed when the culture reached 80–90% confluence, and were harvested by trypsin for the immortalized cell lines, and with the Promocell Detach Kit for the primary osteoblasts per the manufacturer's protocol. All cultures were maintained at 37°C in a humidified atmosphere containing 5% CO2. Prior to experiments, osteoblast cultures were harvested, washed in complete medium, replated in medium containing 2% FCS, and treated as described. Reagents were obtained from the following companies: Recombinant HIV-1_IIIb_ Gp120 (ImmunoDiagnostics, Inc.); Etoposide (Sigma); Insulin (Sigma); TNFα (R&D Systems); skTRAIL (Axxon Life Sciences); AMD3100 (NIH AIDS Research and Reference Program); Pertussis Toxin (CalBiochem); agonistic monoclonal antibody to Fas -CH11- (Millipore); PE labeled anti-human CD4, PE labeled anti-human CXCR4, and PE labeled anti-human CCR5 (BD Pharmingen).

### Cell Viability and Death Assays

Cell viability was measured by exclusion of trypan blue and propidium iodide staining by light microscopy and flow cytometry respectively. Treatment with etoposide (10 µM) was used throughout as a positive control. Briefly, cells treated as described was harvested with trypsin, washed and resuspended in PBS. In the case of trypan blue staining, an aliquot was stained with an equal volume of trypan blue, and live and dead cells counted on a hemocytometer under light microscopy. Percent Viability was calculated as the number of live cells (trypan blue negative) divided by the total number of cells multiplied by 100. For PI staining, cells were resuspended in 1 ml PBS. PI (Sigma) was added to a final concentration of 1μg/ml, and cells were incubated for 30 minutes in the dark prior to flow cytometry analysis on unfixed samples. All flow cytometry was performed on a FACScan (Becton Dickinson) flow cytometer and analyzed with FlowJo software (Version 5.7.2, Tree Star, Inc.).

Hypodiploid DNA, a marker of apoptosis, was assessed as follows. Treated cells were harvested with trypsin, washed, and fixed in 70% ethanol and incubated at −20°C until staining. Cells were thawed, washed in PBS, and resuspended in 1 ml of staining buffer containing 20μg/ml propidium iodide, 0.2 mg/ml DNase-free RNase A (Sigma) and 0.1% (v/v) Triton-X in PBS. Cells were incubated at room temperature in the dark and analyzed by flow cytometry.

Active caspase 3 expression was assessed as follows. Treated cells were harvested, washed and permeabilized in PBS containing 0.2% Tween20 (Sigma) at 37°C for 15 minutes, washed in PBS with 5% FCS, and stained with PE labeled rabbit anti-human active caspase 3 or isotype antibody (BD Pharmingen) at 4°C for 1 hr. Cells were then washed and fixed in 2% paraformaldehyde and analyzed by flow cytometry.

### Cell Proliferation Assays

Cellular proliferation was assayed by measurement of ATP production using the Cell-Titer Glo Luminescent Assay (Promega). Osteoblasts were harvested as above, plated in 96 well microtiter plates at a density of 5×10^5^ cells/well, and allowed to adhere overnight. The following day, the cells were treated as described in 200μl medium in triplicate. At the appointed time, the medium was aspirated, and 50μl Cell-Titer Glo Reagent was added to each well, and incubated in the dark at room temperature for 15 minutes. Luminescence was measured on 10 µl aliquots on a Lumat LB 9501 luminometer. Percent change in ATP content was calculated for each time point as the relative light units emitted per well divided by the mean of the control sample triplicate multiplied by 100.

Cellular proliferation determination by CFSE dilution was measured using the CellTrace CFSE Cell Proliferation Kit (Molecular Probes) according to the manufacturer's protocol. In brief, cells were harvested with trypsin, washed in PBS, and resuspended in PBS with 0.1% BSA at a concentration of 1×10^6^ cells/ml. 1μl of a 5 mM stock CFSE solution in DMSO was added and vortexed. Cells were incubated with dye at 37°C in the dark for 10 minutes, and then staining quenched by addition of 5 volumes ice-cold complete media and incubated on ice for 5 minutes. Cells were washed three times in complete media and then cultured in 12 well plates overnight at 37°C prior to treatment as described. At the appointed time, cells were again harvested with trypsin, washed, co-stained with a PE labeled anti-CXCR4 or isotype antibody as appropriate, washed, and fixed with 2% paraformaldehyde for flow analysis. CFSE dilution was calculated using the division index analysis function in the FlowJo Software.

Intracellular Ki-67 expression was measured via flow cytometry per manufacturer's protocol using FITC-labeled, mouse anti-human Ki-67 (BD Pharmingen). Briefly, cells were treated as described and harvested with trypsin, washed in PBS, and fixed in cold 70% ethanol while vortexing. Fixed cells were incubated at −20°C until staining. For staining, fixed cells were thawed to room temperature, washed in PBS with 1% FCS, and resuspended in 100μl PBS with 1% FCS in a 6 ml FACS tube. 20μl FITC labeled anti-Ki-67 antibody or isotype control was added and cells incubated at room temperature in the dark for 20 minutes. Cells were again washed in PBS and fixed in PBS with 2% paraformaldehyde for flow analysis.

### Statistical Analyses

Values are expressed as mean values +/− standard error, except when indicated otherwise. Comparisons were made using the student's t-test. A P value of <0.05 was considered statistically significant.

## Results

### Recombinat Gp120 does not induce cell death in osteoblast-like cell lines

**Figure 1:**
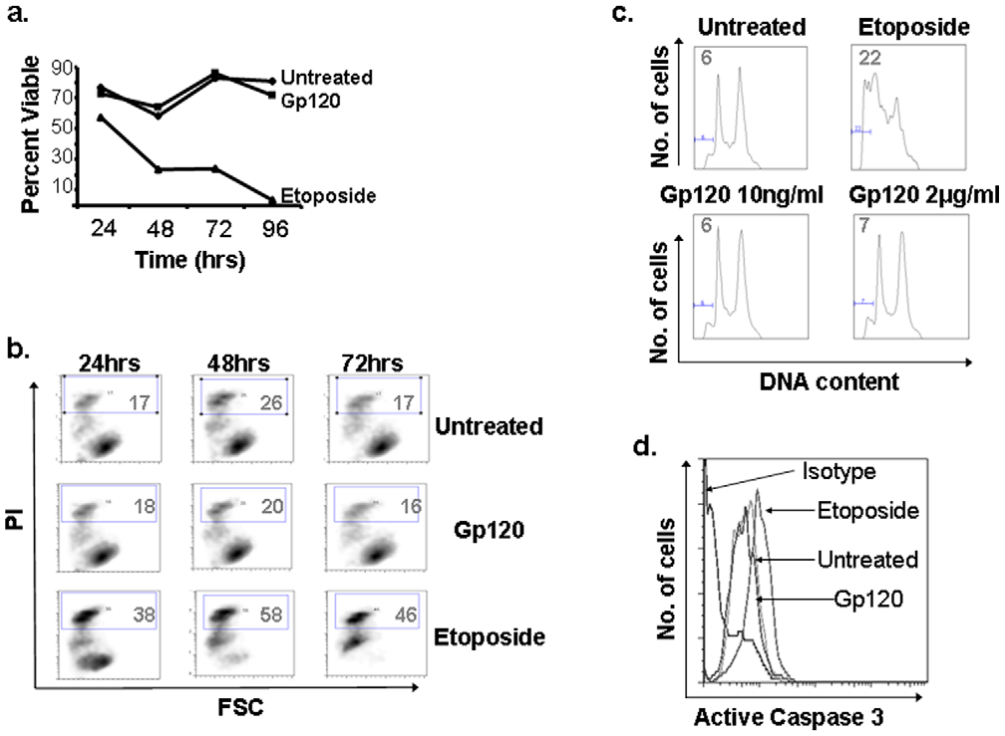
Gp120 does not induce cell death in osteoblasts. FOB cells were cultured in the presence or absence of Gp120 (2μg/ml) in low serum media and assessed for viability by trypan blue exclusion (A), and propidium iodide staining (B). Apoptosis, as indicated by hypodiploid DNA (C) and active caspase 3 expression (D), was assessed by flow cytometry. Etoposide (10μM) was used as a positive control. Results are representative of ≥3 independent experiments.

Because the number of osteoblasts is decreased in HIV-infected patients, and Gp120 has been shown to have pro-apoptotic effects on multiple cell types, we first sought to determine whether Gp120 induces apoptosis in human immortalized fetal osteoblasts (FOB cells) and human osteoblast-like osteosarcoma cells (U2OS cells) that were cultured in the presence or absence of recombinant HIV strain IIIB derived Gp120 over several days. We independently confirmed the biologic activity of the recombinant Gp120 by demonstrating that pre-treatment of Jurkat T cells with Gp120 (1 µg/ml) for 48 hours increased susceptibility to apoptosis induced by T-cell receptor ligation compared to BSA pre-treatment (Supplementary Figure S1). Because Gp120 can be both pro- or anti-apoptotic in similar models at varying concentrations, FOB cells were treated with Gp120 over a wide dose range (100 pg/ml to 5 µg/ml), which did not alter cell viability within 96 hours after treatment (Figure 1A and 1B). Furthermore, Gp120 treatment did not induce DNA hypodiploidy within 48 hours, or the expression of active Caspase 3 within 6 days in FOB cells (Figure 1C and 1D). Similar results were seen with U2OS osteosarcoma cells, and primary human osteoblasts from two HIV-uninfected donors (data not shown). These results indicate that Gp120 itself does not induce apoptotic cell death in these osteoblasts tested.

Because Gp120 has been shown to increase T cell susceptibility to death-ligand induced apoptosis [23], [24], we questioned whether Gp120 treatment would increase osteoblast susceptibility to cell death induced by TNFα, skTRAIL or an agonistic monoclonal antibody to Fas (CH11). However, Gp120 treatment, when compared to untreated controls, did not increase osteoblast cell death in response to TNFα (10 ng/ml), skTRAIL (100 ng/ml) or CH11 (500 ng/ml) (Supplementary Figure S1C) as measured by trypan blue exclusion and ATP content.

**Figure 2:**
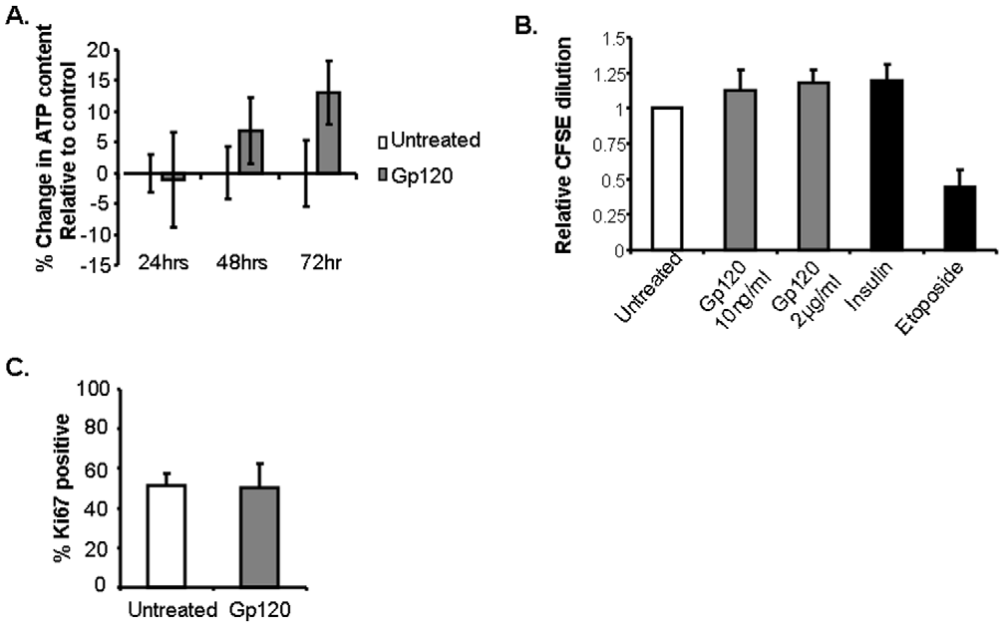
Gp120 does not inhibit osteoblast proliferation. A. FOB and U2OS cells were cultured in the presence or absence of Gp120 (2μg/ml) in low serum media and assessed for proliferation by measuring ATP content as described in Methods. Mean (SEM) values of sample triplicates relative to control are depicted for FOB cells. B. Proliferation in FOB and U2OS cells at 48 hrs was measured by flow cytometry by CFSE dilution (B) and expression of intracellular Ki-67 (C). Mean (SEM) values of 3 independent experiments with U2OS cells are represented.

We next questioned whether, instead of directly or indirectly inducing cell death, Gp120 inhibited proliferation of osteoblasts. FOB and U2OS cells were cultured in the presence or absence of Gp120, and cellular proliferation determined by measurement of cellular ATP content, CFSE dilution and Ki67 expression. Gp120 treatment of osteoblasts did not significantly decrease ATP content (Figure 2A), CFSE dilution (Figure 2B), nor Ki67 expression (Figure 2C), when compared to controls, indicating that Gp120 treatment did not inhibit proliferation of the osteoblast cell lines tested.

### Osteoblast-like cells express the CXCR4 co-receptor, and Gp120 treatment results in proliferation of CXCR4+ osteosarcoma cells

**Figure 3:**
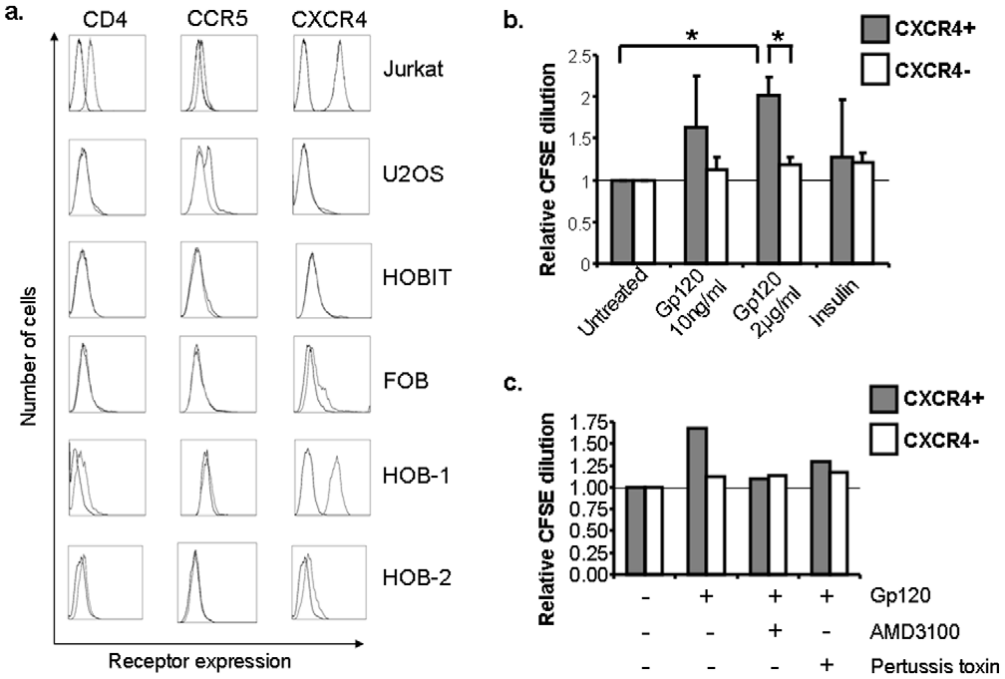
Gp120 induces proliferation in CXCR4 positive U2OS cells. A. Surface expression of CD4, CCR5 and CXCR4 was assessed by flow cytometry in the osteoblast cell lines noted. Jurkat T cells were used as a positive staining control. The darker line represents the isotype antibody control. B. Relative proliferation in CXCR4 positive (CXCR4+) and CXCR4 negative (CXCR4-) U2OS cells was measured by CFSE dilution at 48 hrs. Mean (SEM) division index values of 3 independent experiments relative to untreated controls are represented. * indicates P<0.05. C. U2OS cells pretreated with AMD3100 (5μM) or Pertussis toxin (1μg/mL) for 30 minutes prior to exposure to Gp120 (2 µg/ml), and CFSE dilution was measured at 48 hrs. Representative results from one experiment of three are presented.

Gp120 binds to one or more of several cell surface receptors, including CD4, CCR5 or CXCR4. Human osteoblasts have been shown to express varying levels of all three of these surface receptors [15], [19]. We questioned whether the lack of apoptosis induced by Gp120 in osteoblasts was associated with a lack of surface expression of the cellular receptors. Varying degrees of CXCR4 protein expression on the cell surface were seen in the FOB, HOBIT and U2OS osteoblast cell lines, as well as in two primary osteoblast cell lines (HOB-1 and HOB-2). On the other hand, very minimal CD4 or CCR5 expression was observed, as evidenced by neither a significant shift in the mean channel fluorescence nor a significant identifiably positive staining population for that particular cell type and receptor (Figure 3A).

Because only a small proportion of the osteoblast-like cells expressed a receptor for Gp120 to bind, subtle effects of Gp120 treatment on this subpopulation could be easily missed when accounting for the total population. Therefore, we questioned whether Gp120 treatment may selectively induce apoptosis or inhibit proliferation in osteoblasts expressing CXCR4. By co-staining with a monoclonal antibody to CXCR4, Gp120 did not induce active Caspase 3 expression in CXCR4+ osteoblasts within 6 days after treatment (Supplementary Figure S1D). However, Gp120 treatment did increase CFSE dilution by nearly two-fold by 48 hours in CXCR4+ U2OS cells when compared to untreated cells (P<0.05, Figure 3B). In order to assess the specificity of the Gp120 interaction with CXCR4 (a G-protein coupled receptor [25]), U2OS cells were pretreated with either the CXCR4 antagonist AMD3100 [26] or the G-protein inhibitor pertussis toxin [27]. Pretreatment with both AMD3100 and pertussis toxin abrogated this proliferative effect in CXCR4+ cells, (Figure 3C). These results suggest that Gp120 induces proliferation specifically in CXCR4+ osteoblast-like U2OS cells. Similar results were not seen in the other osteoblast cell lines tested.

## Discussion

In this study, we demonstrate that the HIV envelope protein Gp120, contrary to a single previous report, does not induce cell death in a variety of human osteoblast-like cell lines. Our data also suggest that in a small proportion of osteoblast-like U2OS cells that express CXCR4, Gp120 may actually have a proliferative effect that is G-protein dependent. The pathophysiologic importance of this latter finding in transformed cells is unclear.

There are several potential reasons why our results did not replicate earlier published reports. First, Gibellini et al tested for Gp120 receptor expression on HOBIT cells and primary osteoblasts [19]. They demonstrated minimal (<5%) surface positivity for CD4 and CXCR4, and nearly 15% positivity to CCR5 by flow cytometry, whereas we detected minimal CD4 and CCR5 expression, with widely varying degrees of CXCR4 expression, albeit in different cell lines. Because pre-treatment of the HIV and Gp120 with soluble CD4 abrogated the apoptosis, the authors argued for a CD4 dependent mechanism, despite data from their study and others that demonstrate minimal CD4 expression on osteoblasts [15]. Therefore, it is possible that differences in study findings could be attributable to differences in cell surface expression in cell lines tested. Second, the physiologic level of Gp120 that is actually present in either plasma or tissues in HIV-infected patients remains controversial, and has varied in *in vitro* studies from pM to µM ranges [28], [29]. We tested a wide range of physiologic doses of recombinant Gp120, from the same T-tropic IIIB strain used in previous studies, with no apoptosis-induction seen. Third, in the prior study, only analysis of hypodiploid DNA content was assessed, whereas we assessed cell death and apoptosis by several flow cytometry and non-flow based techniques. Finally, the earlier study contrasts with our data, since the Gp120-induced apoptosis was partially inhibited by treatment with anti-TNF neutralizing antibodies. However, our data suggest that osteoblast like cells are actually resistant to TNFα-induced apoptosis (data not shown), and are consistent with other reports that osteoblasts are relatively resistant to induction of apoptosis by TNF-superfamily ligands, possibly due to expression of non-signal transducing decoy receptors [30] [31], [32].

Gp120 has been shown to induce proliferation in multiple cell types other than osteoblasts *in vitro*. Gp120 induces B cell proliferation and immunoglobulin production [33], and X4 tropic Gp120 increases Epstein Barr Virus-induced transformation in normal B cells [34]. These effects may contribute to both the polyclonal B cell activation and increased frequency of Non-Hodgkin Lymphoma seen in HIV infected patients. Gp120 treatment of vascular smooth muscle cells results in increased proliferation compared to control, possibly through an octapeptide of Gp120 that has sequence homology with neuropeptide Y and signals through the NPY receptor [35]. This effect may contribute to the increased vascular smooth muscle cell hyperplasia and accelerated cardiovascular and microvascular disease seen in clinical HIV infection. Interestingly, Gp120 has bimodal, concentration dependent effects on multiple types of kidney cells, possibly contributing to HIV-associated nephropathy and focal segmental glomerulosclerosis. At lower concentrations, Gp120 induces proliferation of kidney fibroblasts, glomerular epithelial cells, and mesangial cells, whereas at higher doses it induces apoptosis [36], [37] [38]. Postulated signaling mechanisms for the proliferative effects of Gp120 include upregulation of pro-survival transcription factors, including c-fos and c-jun, activation of protein kinase C and tyrosine kinase pathways, and activation of Akt and/or NFκB.

While our data suggest that Gp120 does not have a direct cytotoxic effect on osteoblasts *in vitro*, it is still possible that Gp120 effects bone metabolism *in vivo* through other mechanisms. It may partially counteract the many pro-osteoporosis mechanisms seen in HIV infection, thereby decreasing the observed overall rate of bone loss. Alternatively, Gp120-induced osteoblast proliferation in the short term may contribute to replicative senescence, and exhaustion of the total osteoblast pool, a process which has been implicated in normal age-related bone loss [39]. This is akin to the immune exhaustion of virus-specific CD8 T cells, which, due to persistent antigenic stimulation and chronic immune activation in HIV infection, become anergic and lose proliferative capacity [40]. On the other hand, in one study treatment of primary human osteoblasts *in vitro* with recombinant Gp120 at a dose of 100 ng/mL decreased RUNX2 expression, alkaline phosphatase activity and calcium deposition, as well as increased PPARγ and lipid staining, suggesting that Gp120 may inhibit osteoblast function and promote adipogenesis; similar effects were not seen in mesenchymal stem cells treated with Gp120 in that study.[17]. However, the same group more recently reported that Gp120 treatment of mesenchymal stem cells increases adipogenesis under adipogenic media conditions, and decreases alkaline phosphatase activity in normal media [41]. If Gp120 does indeed upregulate TNFα production by osteoblasts, this could promote osteoclast generation and activity [42]. Gp120-exposed T cells *in vitro* produce RANKL which also stimulates osteoclast generation and activity [10]. Any potential effects that Gp120 might have on osteoblast chemotaxis by competing with the natural ligands for CXCR4 and/or CCR5 would be import to study.

## Supporting Information

Figure S1 A. Jurkat T cells were pretreated with either Gp120 or BSA (1 µg/mL) for 48 hours, and then apoptosis was induced by sequential treatment with 2 µg/ml phytohemagglutinin (PHA) on day 3, 50 U/ml interleukin 2 (IL-2) on day 4, and then 35 µg/ml OKT-3 on day 5. Depicted is mean (SEM) viability by trypan blue exclusion of 3 independent experiments. B. Primary human osteoblasts (HOB-2) were treated with Gp120 (2 µg/ml) or etoposide (positive control), and cell viability measured by trypan blue exclusion. Results are representative of ≥2 independent experiments in two primary human osteoblast cell lines. C. U2OS cells were treated with TNFα (10 ng/mL) alone or with Gp120 (2 µg/ml) and cell viability measured over time by ATP content (see Methods). Depicted results are representative for similar results obtained with skTRAIL and anti-Fas treatment in both U2OS and FOB cells. D. U2OS cells were treated with Gp120 or control, and active caspase 3 expression assessed by flow cytometry in cells expressing CXCR4 and those not expressing CXCR4.
(TIF)
