# Supplementary figures and images for: Human Immunodeficiency Virus Envelope Protein Gp120 Induces Proliferation but Not Apoptosis in Osteoblasts at Physiologic Concentrations
Source: PLoS One. 2011 Sep 12;6(9):e24876. doi: 10.1371/journal.pone.0024876 (PMC3171487; doi:10.1371/journal.pone.0024876)

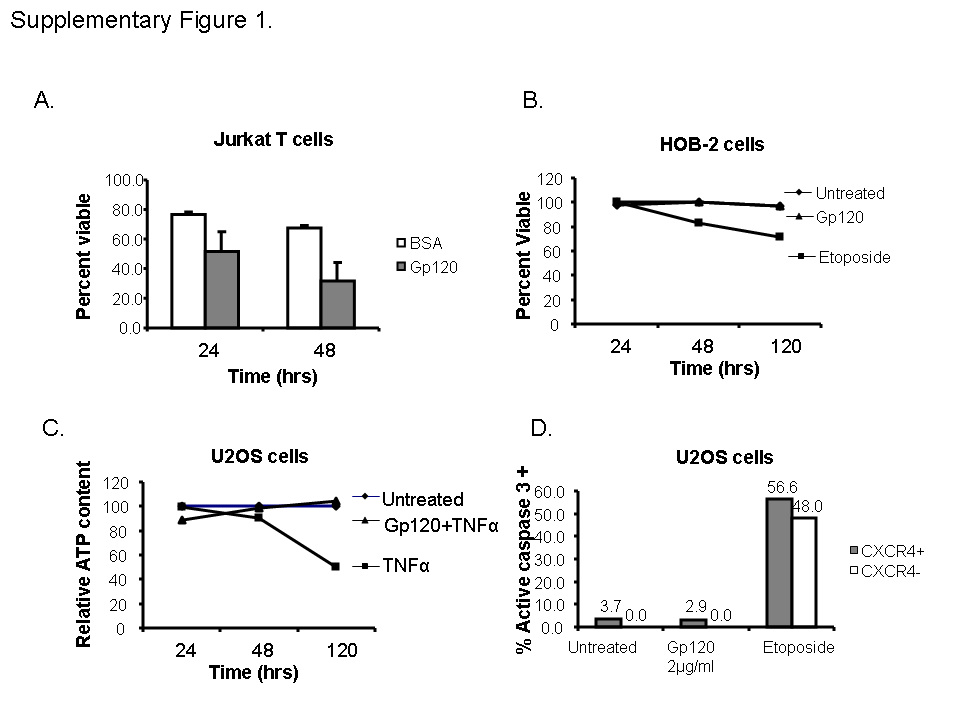

Supplement: Figure S1 — A. Jurkat T cells were pretreated with either Gp120 or BSA (1 µg/mL) for 48 hours, and then apoptosis was induced by sequential treatment with 2 µg/ml phytohemagglutinin (PHA) on day 3, 50 U/ml interleukin 2 (IL-2) on day 4, and then 35 µg/ml OKT-3 on day 5. Depicted is mean (SEM) viability by trypan blue exclusion of 3 independent experiments. B. Primary human osteoblasts (HOB-2) were treated with Gp120 (2 µg/ml) or etoposide (positive control), and cell viability measured by trypan blue exclusion. Results are representative of ≥2 independent experiments in two primary human osteoblast cell lines. C. U2OS cells were treated with TNFα (10 ng/mL) alone or with Gp120 (2 µg/ml) and cell viability measured over time by ATP content (see Methods). Depicted results are representative for similar results obtained with skTRAIL and anti-Fas treatment in both U2OS and FOB cells. D. U2OS cells were treated with Gp120 or control, and active caspase 3 expression assessed by flow cytometry in cells expressing CXCR4 and those not expressing CXCR4. (TIF) [file pone.0024876.s001.tif]
